# Peripheral Demyelinating Diseases: From Biology to Translational Medicine

**DOI:** 10.3389/fneur.2019.00087

**Published:** 2019-03-19

**Authors:** Khidhir Kamil, Muhammad Dain Yazid, Ruszymah Bt Hj Idrus, Srijit Das, Jaya Kumar

**Affiliations:** ^1^Department of Physiology, Faculty of Medicine, Universiti Kebangsaan Malaysia Medical Centre, Kuala Lumpur, Malaysia; ^2^Tissue Engineering Centre, Faculty of Medicine, Universiti Kebangsaan Malaysia Medical Centre, Kuala Lumpur, Malaysia; ^3^Department of Anatomy, Faculty of Medicine, Universiti Kebangsaan Malaysia Medical Centre, Kuala Lumpur, Malaysia

**Keywords:** peripheral demyelinating disease, schwann cell, biomarker, Guillain-Barre syndrome, chronic inflammatory demyelinating polyradiculoneuropathy, anti-MAG neuropathy, POEMS syndrome, Charcot-Marie-Tooth disease

## Abstract

Demyelinating diseases represent a spectrum of disorders that impose significant burden on global economy and society. Generally, the prognosis of these diseases is poor and there is no available cure. In recent decades, research has shed some light on the biology and physiology of Schwann cells and its neuroprotective effects in the peripheral nervous system (PNS). Insults to the PNS by various infectious agents, genetic predisposition and immune-related mechanisms jeopardize Schwann cell functions and cause demyelination. To date, there are no effective and reliable biomarkers for PNS-related diseases. Here, we aim to review the following: pathogenesis of various types of peripheral demyelinating diseases such as Guillain-Barre syndrome, Chronic Inflammatory Demyelinating Polyradiculoneuropathy, Anti-Myelin Associated Glycoprotein Neuropathy, POEMS syndrome, and Charcot-Marie-Tooth disease; emerging novel biomarkers for peripheral demyelinating diseases, and Schwann cell associated markers for demyelination.

## Introduction

Peripheral demyelinating diseases (PDD) refer to a spectrum of disorders that involves substantial damage to axons and glial cells, particularly Schwann cells (SC) in the peripheral nervous system (PNS) (1). The incidence of these diseases is variable (2–4). Disease states are manifestations of damage against the myelin sheath caused by various inciting factors, such as infectious agents, auto-immune processes or genetic mutations (1, 5–7). Oxidative stress, the primary risk factor in many diseases (8), has also been implicated in demyelination disorders (9).

Schwann cells are principal glial cells in peripheral nerves that originate from the neural crest, which is a multipotent embryonic structure that also differentiates into other main glial subtypes of the PNS (10). SC development occurs through a series of embryonic and postnatal phases, which are tightly regulated by a number of cellular signaling pathways. During the early embryonic phase, neural crest cells differentiate into SC precursors that represent the first transitional stage in the SC lineage, that subsequently further differentiate into immature SC (10). At time of birth, these immature SC differentiate into either myelinating or non- myelinating SC that populates the mature nerve trunks and wrap around axons through a process known as myelination (10).

Myelination is a process whereby SC develops a multi-layered membrane called the myelin sheath around the axonal membrane (11). Mostly, larger axons (>1 um) are selected specifically by SC to form multiple internodes of the myelin sheath (12). Myelination begins with the establishment of a 1:1 relationship with the axon. At this level, the production of myelin structural proteins such as myelin protein zero (P0), peripheral myelin protein (PMP22), myelin basic protein (MBP) are increased along with lipid biosynthesis (11).

The myelin sheath is made of multiple sleeves of whitish lipoprotein plasma membranes of SC wrapped around the axon of a neuron in a spiral fashion (13). It is constituted of water, lipid and proteins that exist as segmented internodal structure around the axons (13). These internodes create insulation that facilitates propagation of action potentials by mean of saltatory conduction (jumping) at the node of Ranvier. Myelin sheath not only facilitates the conduction velocity of nerve impulse but also confers protection and nutritional support to axons. However, exposure to various factors such as autoimmunological insult, trauma, and injury to the nerve could trigger demyelination, and eventually neurodegeneration (14).

## PNS Demyelinating Diseases

Demyelination describes the loss of the myelin sheath, where SC are being destroyed or unwrapped from axons (15). Demyelination causes neurological disability due to conduction block and axonal degeneration. Diagnosis of PDD depends on electrophysiological and cerebrospinal fluid (CSF) analysis. However, in some cases, no biomarkers are clinically available for diagnosis, disease monitoring and prognosis.

### Acquired Demyelinating Disease

#### Guillain-Barre Syndrome

Guillain-Barre Syndrome (GBS) is an acute idiopathic autoimmune demyelinating disease of the PNS that is characterized by acute flaccid ascending neuromuscular paralysis (16). GBS is rare, with an incidence of 0.8–1.9/100,000 per annum across Europe and North America (17). Currently, the specific causative agent of GBS is unknown, but numerous theories have been proposed (18). Most cases of GBS are preceded by antecedent infections of several microbes of the gastrointestinal and upper respiratory tracts (19). Among those, 60% of GBS cases were related to autoantibodies, anti-monosialotetrahexosylganglioside- 1 (anti-GM1) and anti-ganglioside GD1a (anti-GD1a) associated with C. *jejuni* infection (20). Other microbes involved include M. *pneumoniae*, cytomegalovirus, Epstein-Barr virus, varicella zoster virus, and influenza virus (21–24). Apart from infections, some GBS cases are results of trauma, surgical interventions, treatment with monoclonal antibodies and vaccination (rare) (20).

**Figure 1:**
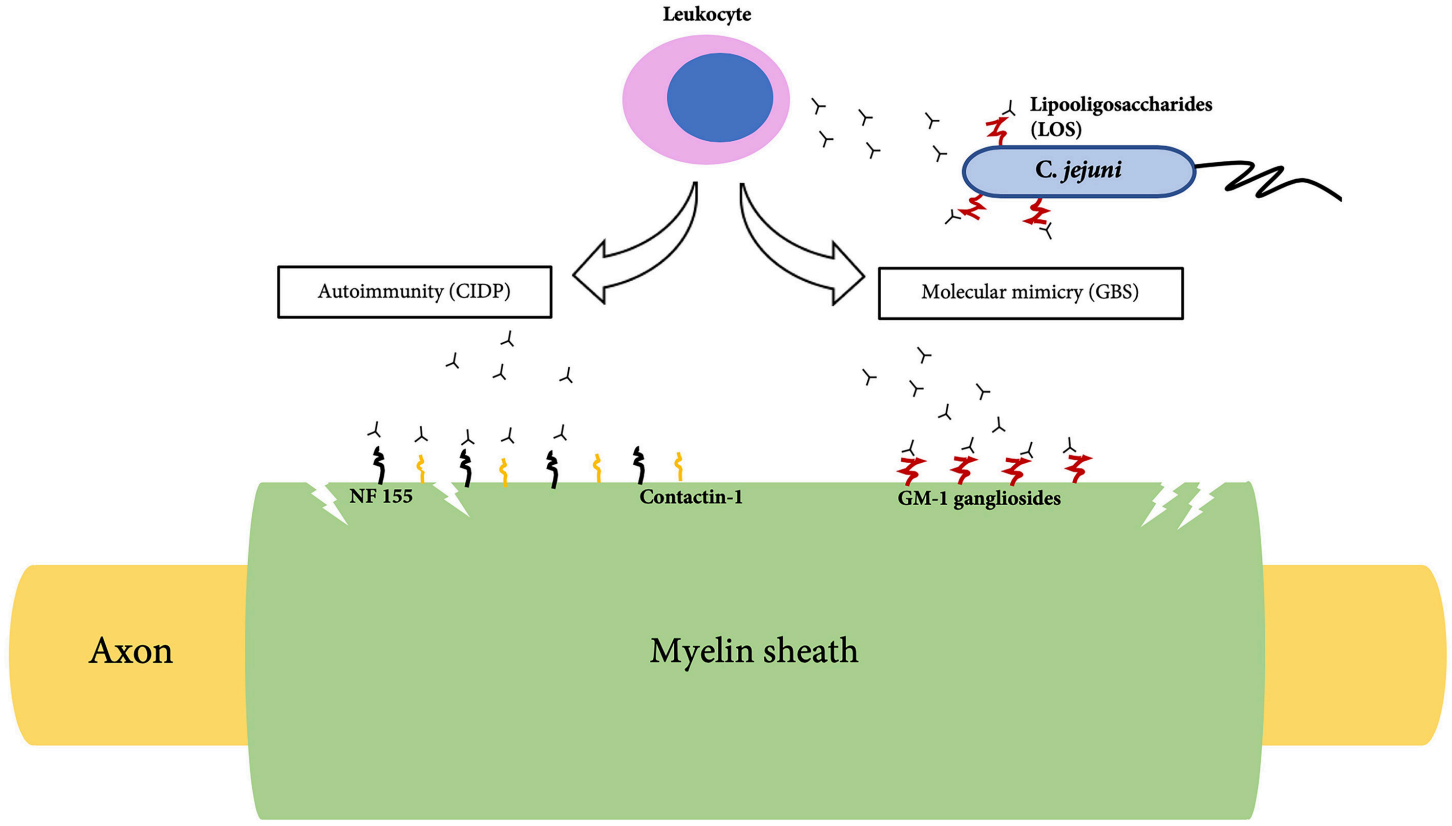
Pathogenesis of Guillain-Barre syndrome (GBS) and Chronic inflammatory demyelinating polyradiculoneuropathy (CIDP).

The most frequent GBS variant is acute inflammatory demyelinating polyradiculopathy (AIDP); other axonal variants include acute motor axonal neuropathy (AMAN), acute motor sensory axonal neuropathy (AMSAN), Miller-Fisher syndrome and oropharyngeal weakness (25, 26). In AIDP, there are areas of segmental demyelination with inflammatory infiltrates such as lymphocytes and macrophages (27). Due to the observation of a high incidence of GBS after a preceding infection, it has been theorized that molecular mimicry plays a role in triggering an autoimmune response against peripheral nerve tissues. The presence of anti-GM1 and anti-GD1a antibodies in the serum suggest that ganglioside-like moieties carried by lipooligosaccharides found in the bacterial wall of C. *jejuni* has cross-reactivity against neural tissues of the PNS (5) (Figure 1). It has also been found that patients treated with gangliosides for pain and neuropathy in the early 1990s later developed GBS (28). Gangliosides, axo-glial junctional proteins, neurofascin and gliomedin at nodes of Ranvier could contribute toward the autoimmunity seen in GBS (29).

The clinical manifestations of GBS include acute ascending fairly symmetric paralysis and paresthesia, choking and difficulty in breathing over the course of hours to several days (2). Involvement of the respiratory muscles in GBS may require the need for artificial ventilation (30). Some patients also experienced autonomic dysfunctions such as cardiac arrhythmia, arterial hypotension, gastrointestinal dysmotility, urinary retention, and abnormal sweating (31). Management of GBS is mostly supportive (20). Affected patients would require comprehensive assisted respiratory ventilation with monitoring for cardiac arrhythmia and bed-bound complications such as ventilator-associated pneumonia, thromboembolism and infections (32). Plasma exchange and intravenous immunoglobulin (IVIG) have been shown in large randomized trials to be beneficial (33). Overall, most cases of GBS have good prognosis with functional recovery within 12 months after disease onset (34). However, some patients do suffer from residual deficits (35).

#### Chronic Inflammatory Demyelinating Polyradiculoneuropathy

Chronic inflammatory demyelinating polyradiculoneuropathy (CIDP) is an acquired immune mediated demyelinating disease of the PNS characterized by progressive loss of motor and sensory functions (36). CIDP sometimes is quite similar to GBS, with the distinction that its clinical course is chronic with relapses (37). The onset is insidious and occurs more commonly in older age individuals (38, 39).

The immune system primarily attacks and damages the myelin sheath of the PNS followed by segmental demyelination and axonal degeneration (6). Histological findings of CIDP demonstrate thin myelin sheath with short internodes described as onion bulbs. Demyelination is indicated by the slow nerve conduction velocity suggestive of conduction block (6). Recently evidence of autoimmunity toward neurofascin-155 (NF155) and contactin-1 (CNTN1) in some patients have been reported. (40, 41) (Figure 1). NF155 is an adhesion molecule that is expressed at paranodes of glial side which interacts with CNTN1, a key axonal adhesion molecule (42). This interaction is essential for the formation of paranodal septate-like junction and loss of this junction is associated with slow conduction (42).

Symptoms of CIDP develop slowly but progressive and neurological deficits peak after 8 weeks of disease onset (36). Typical symptoms are tingling/numbness of the extremities due to the association of large nerve fibers, symmetrical weakness and paresthesia of legs and arms, loss of reflex, fatigue, ataxia and limb incoordination (6). Treatment with oral glucocorticoids usually produce a favorable response (43). Apart from that, plasmapheresis and IVIG are also effective (36).

#### Anti-Myelin Associated Glycoprotein (MAG) Neuropathy

Anti-Myelin Associated Glycoprotein (MAG) neuropathy is a demyelinating polyneuropathy associated with IgM monoclonal gammopathy towards MAG in peripheral nerves (44). MAG is a type I transmembrane glycoprotein l presents in peri-axonal SC and oligodendroglial membranes of myelin sheaths that central in glial-axon interaction and maintenance of axonal function (45). Loss of MAG compromises the myelin sheath integrity and axonal function. MAG contains a carbohydrate epitope shared with other glycoconjugates that serve as primary antigenic targets for IgM paraproteins (44). Injection of serum containing IgM anti-MAG paraproteins into chickens causes segmental demyelination and conduction block (46).

The disease is also described as progressive mild to moderate distal muscle weakness; along with progressive sensory ataxia and frequent tremors (47). The clinical course is generally benign, with minimal functional deterioration manifested over time (47). As the symptoms of anti-MAG neuropathy usually are minimal and do not interfere with the patient's daily activities initially; management at this stage comprises of supportive care such as exercise and balance training. However, patients with sensorimotor weakness should be treated. Steroids, IVIG and plasmapheresis are rarely effective. Rituximab, a monoclonal antibody against CD20 surface antigen is promising (48).

#### POEMS Syndrome

POEMS syndrome is a rare paraneoplastic syndrome with demyelinating neuropathy (49). Emprical data on POEMS syndrome is deficient owing to the complexity and multisystemic nature of its clinical manifestations. It is usually associated with an underlying plasma cell neoplasm (50). POEMS syndrome commonly presents in the fifth to sixth decade (49). The pathogenesis of POEMS syndrome is not well understood, but several hypotheses have been proposed. High serum level of vascular endothelial growth factor (VEGF) is detected in POEMS patients, whereas low levels are often reported upon successful treatment (50). The pathological assessment does not reveal inflammatory infiltrates or immunoglobulin deposition within the nerves; instead, there is endothelial cell hypertrophy with extended process, reduced luminal diameter, and disrupted tight junction that could cause leakage (51). Excessive VEGF secreted by plasma cells is thought to cause endothelial proliferation and subsequent leaky vessels that compromise blood flow (51).

POEMS is an acronym of its multiorgan features: Polyneuropathy, Organomegaly, Endocrinopathy, M protein, and Skin changes. The polyneuropathy involves both sensory and motor systems (52). Patients usually begin to experience sensory abnormalities described as tingling, paresthesia and coldness in the feet, along with touch, pressure, and proprioception disturbances (52). Motor symptoms then develop, including symmetrical severe weakness on extremities progressing distally with gradual spreading to proximal (52). Nerve conduction studies, as well as nerve biopsies show evidence of demyelination and axonal loss (49). Hepatomegaly is commonly reported, but splenomegaly and lymphadenopathy are not frequent. Endocrinopathy, usually gonadal dysfunction is noted by testicular atrophy and gynecomastia and diabetes mellitus. The M-protein of IgG or IgA is commonly detected. Skin changes include hyperpigmentation and hypertrichosis. Other additional features that are not included in the acronym are sometimes present. These include peripheral edema, effusion in body cavities such as ascites, sclerotic bone lesions, Castleman's disease, elevated intracranial pressure, papilledema, fatigue, renal failure and clubbing (50). However, not all features are necessarily required for the diagnosis.

Treatment with high dose chemotherapy and autologous peripheral blood stem cell transplant are the first line therapy. Alternative therapeutics are including corticosteroids, low-dose alkylator therapy and radiation therapy. The median survival for patients with POEMS syndrome is about 13.8 years. Mortality usually results from cardiorespiratory failure, infection and renal failure. Supportive care such as physical and occupational therapy should be in line with the treatment to improve outcome and quality of life. Some patients might even require assisted ventilation due to respiratory muscle weakness (49).

### Inherited Demyelinating Disease

#### Charcot Marie Tooth Disease

Charcot Marie Tooth disease (CMT) is a rare hereditary neurological disorder affecting the peripheral nerves (7). Although CMT is rare, it is the most commonly inherited form of neuropathy affecting approximately 1 in 2,500 people (53). The majority of CMT have an autosomal dominant inheritance but X-linked and autosomal recessive pattern also exist (54). The gene abnormalities in CMT disrupt the structure and functions of Schwann cell and peripheral nerve axons. Several subtypes of CMT have been identified: mutations of genes encoding myelin-related proteins such as PMP22, P0, and connexin 32 are classified as demyelinating subtype (54); mutations of proteins involved in axonal transport such as mitofusin-2(MFN2), ganglioside-induced differentiation- associated protein 1 (GDAP1), heat shock factor binding protein 1 are classified as axonal neuropathies subtype (54). Next-generation gene sequencing involving 17,000 samples with neuropathy identified the prevalence of specific mutations as such: 78.6% involve PMP22 mutations, 6.7% involve GJB1, 5.3% involve P0, and 4.3% involve MFN2 (55).

The most common form of CMT, CMT1A is a result of duplications of the PMP22 gene that increased expression of PMP22 structural protein (56). CMT1B on the other hand, involves mutations of P0 gene that result in misfolding and retention of a mutant P0 protein intracellularly (54). This condition triggers the activation of unfolded protein response which later lead to cell apoptosis (57). CMT2A involve mutations of MFN2 which aids in the fusion of mitochondria (54, 58). Typically, the progression of CMT is slow. The neuropathy of CMT could affect both motor and sensory nerves. Patients may experience distal muscle weakness, foot drop that formed pes cavus, scoliosis and hammer toes. Respiratory insufficiency is rare, but possible. Neuropathic pain and fatigue have been reported in several cases.

At present, the management of PDD is central upon synthetic drugs and natural products (59). However, this disease remains underdiagnosed owing to lack of reliable biomarkers and a disease specific-diagnostic criteria.

Table 1 summarized the pathogenesis, clinical features and management of PDD.

**Table 1:** Pathogenesis, clinical features and management of various types of Peripheral Demyelinating diseases.

	**Etiology**	**Risk factors**	**Pathology**	**Clinical features**	**Management**
**ACQUIRED DEMYELINATING DISEASE**					
Guillain-Barre syndrome	Unknown	Antecedent infections: C. jejuni, M. pneumoniae, cytomegalovirus, Epstein-Barr virus, varicella zoster virus Trauma Surgery Vaccination	Segmental demyelination Inflammatory (macrophage, lymphocyte) infiltrates	Acute ascending symmetric paralysis, paresthesia, choking, difficulty in breathing, autonomic dysfunctions (hours to several days)	Supportive therapy Plasma exchange IVIG
Chronic inflammatory demyelinating polyradiculoneuropathy	Unknown	Autoimmunity	Segmental demyelination Thin myelin sheath Onion bulb (short internodes) formation Perivascular inflammatory infiltrates	Slow, progressive neurological deficits such as tingling, numbness, symmetrical weakness of limbs, paresthesia of limbs, loss of reflex, ataxia, limb incoordination. (slow, progressive)	Glucocorticoids Plasmapheresis IVIG
Anti-Myelin Associated Glycoprotein (MAG) neuropathy	MAG IgM monoclonal gammopathy		Segmental demyelination, Immunoglobulin deposits	Benign, minimal distal muscle weakness, progressive sensory ataxia, tremors. (several years)	Supportive therapy (exercise, balance training) Corticosteroids, IVIG, Plasmapheresis (rarely effective)
POEMS syndrome	Unknown (paraneoplastic syndrome)	Plasma cell neoplasm	Endothelial cell hypertrophy with disrupted tight junction No inflammatory infiltrates No immunoglobulin deposition	Polyneuropathy (paresthesia, motor weakness, sensory disturbance), organomegaly (hepatomegaly, lymphadenopathy), endocrinopathy (testicular atrophy, gynecomastia), paraproteinemia (M-protein), skin changes (hyperpigmentation, hypertrchosis), peripheral edema	High dose chemotherapy Stem cell transplant Corticosteroids Alkylator therapy Radiation therapy Supportive therapy
**INHERITED DEMYELINATING DISEASE**					
Charcot Marie Tooth disease	Mutations of genes (PMP22, P0, connexin 32, mitofusin-2, etc.)		Segmental demyelination Onion bulb formation	Distal muscle weakness, foot drop, scoliosis, hammer toes, neuropathic pain, fatigue, sensory disturbance (slow, not progressive)	Supportive therapy (exercise, muscle training, balancing)

## Emerging Biomarkers of Peripheral Demyelinating Disease

According to the National Institute of Health Biomarkers Definitions Working Group, biological marker (biomarker) is defined as “a characteristic that is objectively measured and evaluated as an indicator of normal biological processes, pathogenic processes, or pharmacological responses to a therapeutic intervention” (60). Biomarkers serve as an important clinical tool in disease diagnosis, therapeutic response and prognosis (60).

To identify the recent progress in biomarkers of PDD, we have conducted a literature search from SCOPUS search engine and database on biomarkers of distinct PDD from the year 2014–2018. Only original articles and in the English language were selected to be included in the review (Table 2).

**Table 2:** Recent biomarkers of Peripheral Demyelinating diseases.

**Disease**	**Biomarkers**	**References**
Guillain-Barre Syndrome	Neutrophil-lymphocyte ratio	(61) (62) (63)
	Platelet-lymphocyte ratio	(61)
	Monocyte-lymphocyte ratio	(63)
	Serum IgG	(64)
	Piccolo protein	(65)
	Anti-ganglionic nicotinic acetylcholine receptor	(66)
Chronic inflammatory demyelinating polyradiculoneuropathy	Neurofascin-155	(67)
	Contactin-1	
	Contactin-1/Contactin-associated protein 1/2	
	P_0_, PMP22	
	Neuronal cell adhesion molecule	
	Gliomedin	
	Subunit of sodium channel at node of Ranvier (NavB1, NavB2)	
	Serum IgG-Fc sialylation	(68)
Anti-MAG neuropathy	anti - SGPG	(69)
POEMS syndrome	Vascular endothelial growth factor (VEGF)	(50)
	Serum free light chain	(70)
	Serum heavy/light chain	(70)
Charcot-Marie Tooth disease	PMP22, P_0_, MFN2, GJB1 mutations	(55)

*Articles were selected from 2015-2018 from SCOPUS database*.

### Biomarkers of GBS

Traditionally, the diagnosis of GBS has relied on clinical features such as electrodiagnostic studies and CSF analysis. Testing for serum IgG antibodies to gangliosides Q1b (GQ1b) is available and useful for the diagnosis of GBS variants (Miller Fisher syndrome) with 85 to 90 percent sensitivity although it is not routinely indicated^1^. Biomarkers for GBS have negligible clinical value, low sensitivity/specificity and costly. Furthermore, the laboratory measurement standard for these biomarkers also has not been established and studies using different methods have been plagued by inconsistent findings (71).

At present, novel biomarkers are being explored for better prognosis of GBS patients. The likes of neutrophil-lymphocyte ratio (NLR) and platelet-lymphocyte ratio (PLR) has received much attention as novel prognostic biomarkers of inflammation. Analysis of NLR and PLR levels of 62 GBS patients prior to-and following intravenous immunoglobulin treatment (IVIG) revealed NLR as a better prediction tool for the acute period of AIDP (major variants of GBS) with 83% sensitivity and 93% specificity. Whereas, PLR only showed 74% sensitivity and 70% specificity (61). NLR also studied for its correlation with the degree of weakness in several muscles assessed through the Medical Research Council (MRC) score (62). High levels of NLR were seen in the lower MRC score upon admission and increased baseline disability among GBS patients (62).

More recent findings indicate monocyte-lymphocyte ratio (MLR) along NLR as a better prognostic marker for GBS (63). NLR and MLR are significantly higher in GBS patients compared to healthy controls. Moreover, NLR and MLR are found to be tremendously increased in severe group (63). On the other hand, Piccolo, a multidomain zinc finger protein that is involved in synaptic active zones and synaptic vesicle trafficking was shown to present in sera of GBS patients. High serological levels of Piccolo were associated with better outcomes in GBS patients (65).

Ganglionic nicotinic acetylcholine receptors (gAChR) are nicotinic receptors that assist synaptic transmission in peripheral autonomic ganglia. Over the last decades, the autoantibodies toward gAChR have been associated with autoimmune dysautonomia (72). Several GBS patients experience autonomic dysfunctions such as cardiac arrhythmia and urinary retention (31). Detection of autoantibodies against gAChR could measure the risk of developing debilitating autonomic dysfunction. High level of α3 or β4 subunits of gAChR was detected in 13.6% of GBS patients with autonomic symptoms through luciferase immunoprecipitation (66).

### Biomarkers of CIDP

Proteomic analysis of the CSF was used to isolate potential biomarker target specific to CIDP (73). Unfortunately, the disease specificity of the identified protein was low. The captured proteins include transferrin, proapolipoprotein, retinal binding protein and transthyretin (73). Several autoantibodies, including NF155, CNTN1, and contactin-1/contactin-associated protein 1 (CNTN1/CASPR1) complex also have been associated with the pathogenesis of CIDP (67).

Autoantibodies against ganglioside antibodies, myelin protein (P0, PMP22), nodal proteins (NF155, CNTN1, CNTN1/CASPR1, CNTN2/CASPR2 complex, neuronal cell adhesion molecule (NCAM)), gliomedin and two subunits of sodium channel at nodes of Ranvier (NavB1, NavB2) have been investigated in CIDP patients. Among these patients, 11 expressed anti-ganglioside antibody reactivity (anti-GM1, anti-GD1b), 4 reacted to CNTN1 (6.2%), 3 reacted to NF155 (6.2%), 1 against CNTN1/CASPR1 complex (1.5%) and 1 against PMP22 (67).

Current literature also associates reduced sialylation of IgG-Fc with increased clinical severity of CIDP. Sialylation and galactosylation of IgG-Fc were significantly lower in CIDP patients. Treatment with IVIG, on the other hand, increased the levels of sialylated IgG-Fc and concurrently attenuated the disease severity (68).

### Biomarkers of Anti-mag Neuropathy

The diagnosis of Anti-MAG Neuropathy is through the detection of autoantibodies against MAG. Early findings from Latov's laboratory detected anti-MAG IgM in the sera of almost more than half of the anti-MAG patients (74). Some anti-MAG IgM also co-reacted with acidic glycolipid in the ganglioside fraction of the peripheral nerves, GM1, GD1a and were identified as sulfoglucuronyl glycosphingolipid (SGPG) (69). In clinically indistinguishable anti-MAG neuropathy without seropositivity of MAG and SGPG, the IgM may react to gangliosides such as GD1b, GT1b, and GQ1b (75, 76). Anti-MAG titers could also be correlated with prognosis of the neuropathy. High baseline and increasing anti-MAG titer correlated with higher chances of recurrence (77).

### Biomarkers of POEMS Syndrome

VEGF, an angiogenic factor, is markedly elevated in POEMS syndrome patients and also referred as one of the major criteria for diagnosis of POEMS syndrome (50). Clinical improvement and prolonged relapse-free survival were reported among patients with normalized serum level of VEGF (78).

### Biomarkers of Charcot-Marie Tooth Disease

Diagnostic markers for CMT disease are usually determined through genetic testing. Once the suspicion of CMT disease is made through clinical judgement and electrophysiological studies.

Diagnosis is confirmed through testing for mutations on PMP22 (CMT1A, most common), GJB1 (CMTX1), P0 (CMT1B), and MFN2 (CMT2A).

## Myelin Sheath-Associated Markers

**Figure 2:**
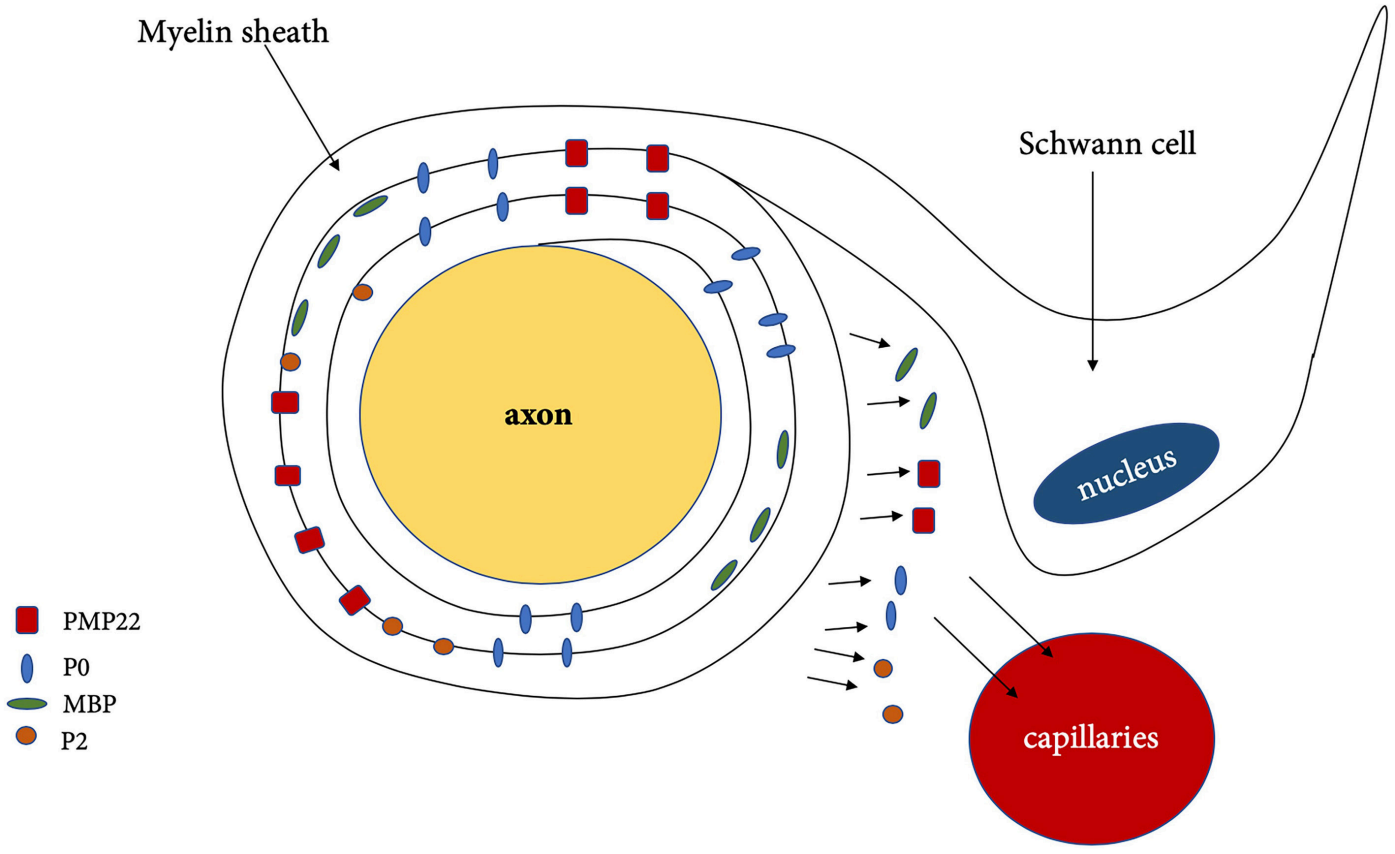
Demyelination and released of myelin associated protein. PMP22, peripheral myelin protein 22; P0, myelin protein zero; MBP, myelin basic protein; P2, myelin protein 2.

During demyelination, various components of myelin and axon are being released as a result of the damage toward the Schwann cells. Myelin sheath consists of approximately 70 % of lipids such as gangliosides, phospholipids, sphingomyelin and 30% of proteins such as myelin [P0, PMP22, myelin protein 2 (P2)] and nodal proteins (neurofascin, gliomedin, contactin) (Figure 2) (Table 3) (13). These components can be detected in CSF, serum and even peripheral nerve biopsies (71), which can potentially indicate the degree of demyelination during the disease progress and also the efficacy of the treatment as reflected by the degree of remyelination.

**Table 3:** List of myelin associated biomarkers.

	**Markers**	**Disease**	**Sample**	**References**
Myelin sheath lipid markers	LM-1, Hex-LM1, GT1b, SGPG	GBS	Serum	(79) (26, 80)
	GD1a, GD1b	GBS	Serum	(81)
	PC, PI, PG, PS, PE, PA, cardiolipin	GBS	Serum	(82)
	Sphingomyelin	GBS, CIDP	CSF	(83)
Myelin sheath protein markers	P_0_	GBS, CIDP	Serum	(84)
		CIDP	Serum	(85)
		CIDP	Serum	(86)
		GBS, CIDP	Serum	(87)
		GBS, CIDP	Serum	(88)
		GBS, CIDP	Serum	(89)
		GBS	Serum	(90)
	P2	GBS, CIDP	Serum	(84)
		GBS, CIDP	Serum	(89)
	PMP22	CMT1A, CMT2, CIDP, anti-MAG, Miller Fisher Syndrome	Serum	(91)
		GBS, CIDP	Serum	(92)
		GBS, CIDP	Serum	(87)
Myelin sheath nodal protein markers	Paranodin	CIDP	Biopsy	(93)
	NF186, gliomedin, NCAM	GBS, CIDP	Serum	(94)
	NCAM	PDN, axonal PN, non-inflammatory diabetic PN	Serum	(95)

Glycolipids such as gangliosides are one of the major lipid components of the myelin sheath. Situated in the plasma membrane with the hydrophilic carbohydrate moiety exposed extracellularly, gangliosides have a greater propensity for autoimmune reaction, especially in GBS, CIDP, and anti- MAG neuropathy (26). Thus, detection of antibody towards gangliosides such as anti-LM1, anti-Hex-LM1, anti-GT1b, anti-SGPG, anti-galactocerebroside in some GBS patients could serve as a diagnostic tool. In parallel to this, the presence of antibodies towards ganglioside complex (GD1a/GD1b, GD1b/GT1b) in serum has been associated with better prognosis of GBS in terms of disease severity (81).

Association of autoimmune diseases such as systematic lupus erythematosus, scleroderma with GBS (96) led researchers (Nakos et al) to ascertain the role of few antiphospholipid antibodies which include phosphatidic acid (PA), cardiolipin, phosphatidylethanolamine (PE), phosphatidylcholine (PC), phosphatidylserine (PS), phosphatidylinositol (PI), phosphatidylglyecerol (PG), and cardiolipin. The antibody levels were shown to decrease following 1-day treatment with γ-globulin (IVIG) and increased 2 days following cessation of treatment (82). This indicates that serum levels of anti-PI and anti-cardiolipin antibodies may be useful to monitor the response of the patient toward treatment with IVIG in GBS patients (82).

Sphingomyelin, another myelin-enriched lipid was reported to be significantly higher among PDD patients (GBS and CIDP) (83). The ability to distinguish between demyelinating variant from axonal variant supports the connotation that sphingomyelin is specific biomarker for peripheral myelin breakdown. In addition, the techniques used to detect and quantify sphingomyelin were also reliable, cost-effective with good sensitivity and specificity (83).

**Figure 3:**
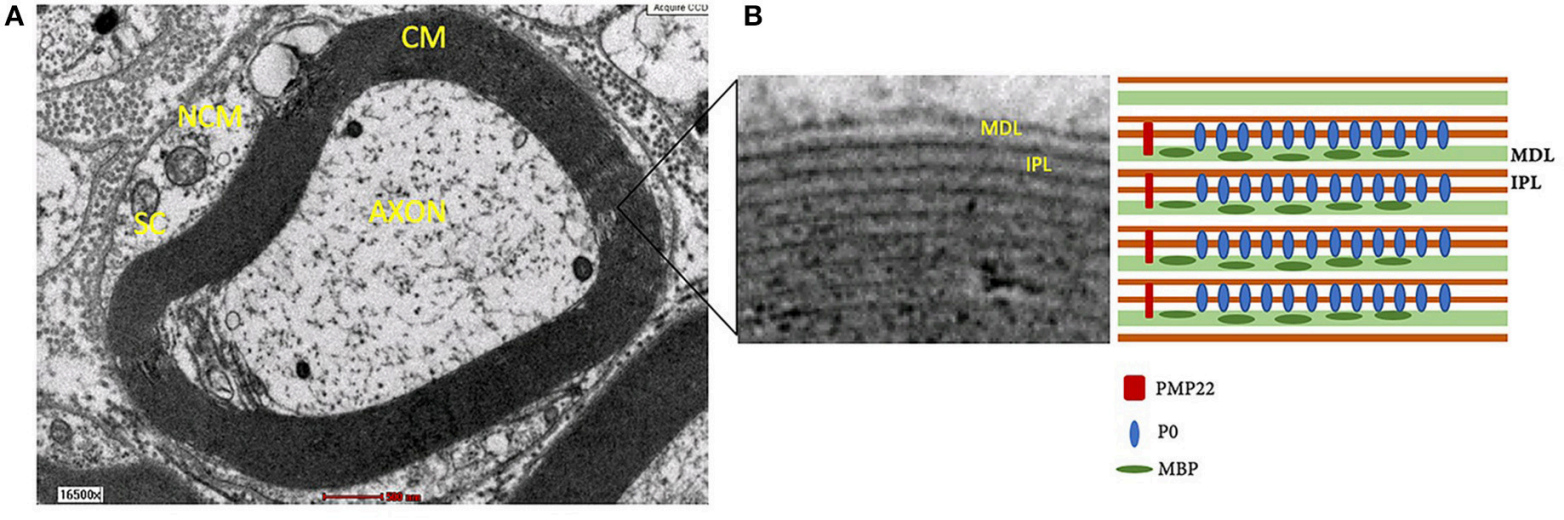
**(A)** Structure of myelin sheath of Schwann cell around axon under electron microscope. **(B)** Compact myelin that consist of major dense line and intraperiod line. NCM, non-compact myelin; CM, compact myelin; SC, Schwann cell; MDL, major dense line; IPL, intraperiod line; P0, myelin protein zero; MBP, myelin basic protein; PMP22, peripheral myelin protein 22.

Although the lipid/protein ratio and lipid constituents of myelin sheath in both CNS and PNS are similar, the distribution and type of myelin proteins in PNS are different. Myelin sheath consists of two compartments, compact myelin (dense area around axon) and non-compact myelin (Figure 3). The compact myelin consists of intraperiod line and the major dense line (MDL). The myelin- specific protein in PNS includes P0, PMP22, and P2.

P0 is a transmembrane glycoprotein that stabilizes the intraperiod line through homophilic binding to another P0 protein (97). Knockout P0 mice were shown to undergo severe hypomyelination and also demonstrated thin, non-compacted myelin sheath with axonal degeneration. In addition, the mice also exhibited tremors, convulsion and deficits in motor coordination (98). P2 protein, also participate in fusion of the MDL in compact myelin (99). PMP22 is a transmembrane protein (100, 101) that is synthesized by Schwann cells and makes up 2–5% myelin protein (102).

Myriad studies have reported the development of antibodies toward P0, PMP22, and P2, especially among GBS and CIDP patients (84, 86–88, 92). The antibodies toward P0 were only present in small proportion of patients with GBS and CIDP and therefore were not useful as a diagnostic test (84). Antibodies toward P0 were only detected in 22% of CIDP and 19% of GBS patient (88). Other studies also reported lower levels of P0 antibodies using various techniques including ELISA, Immunoblot, and Western blot (85, 86, 89). In contrary, some studies reported absence of immune response or no significant response toward P0 (87, 90).

Compared to P0 antibodies, detection of P2 antibodies was reported to be more common in some cases of GBS and CIDP (84). Subsequent studies revealed trace level of IgG to anti-P2 in GBS patients during the peak stage of the disease. The detected trace level of P2 in most patients with GBS and CIDP across all stages of the diseases were within the same range as in the control group (84, 89). Therefore, P2 is not sensitive and specific biomarkers for demyelinating diseases.

PMP22 was detected in 70% of CMT1 and 60% of CMT2 cases. Surprisingly, no significant difference in the immune response of PMP22 from healthy donors and patients with acquired neuropathies (91). Similarly, another study also reported antibodies against PMP22 in 52% of GBS and 35% of CIDP patients (92). Contrary to these findings, absence of an immune response to PMP22 P0 and Cx32 proteins were reported in GBS and CIDP patients (87). These discrepancies were probably due to different usage of PMP22 antigen and also different patient groups that might reflect distinct immunoreactivity toward PMP22.

## Myelin Nodal Protein

**Figure 4:**
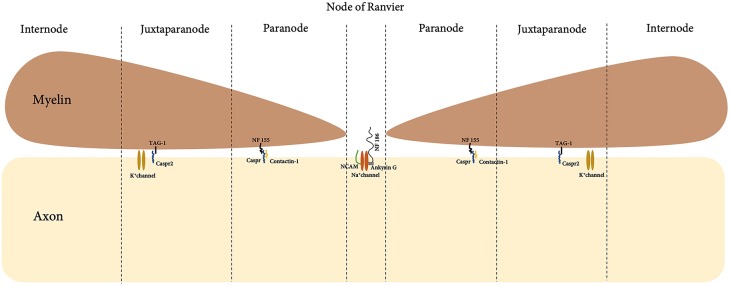
Anatomical compartment of the node of Ranvier and myelin.

In a myelinated fiber, there are multiple internodes of Schwann cells, which are separated by the node of Ranvier. A detailed and magnified look into the boundaries of the node of Ranvier with Schwann cell internode further shows detailed compartment that viewed the node, paranode and juxtraparanode (Figure 4). Nodal proteins such as contactin, neurofascin and NCAM were identified at these regions and they took part in the formation of the septate-like junction that closely in contact with axons (67).

Abnormalities of the nodal proteins were reported in the pathogenesis of CIDP (103). Paranodin, a nodal protein involved in axoglial contacts was tested through immunostaining of biopsies from patients with CIDP. Evaluation of positively-stained paranodin biopsies led to the correct diagnosis of CIDP in 70% of the reported cases (93). Further laboratory investigations revealed IgG autoimmunity towards myelin nodal proteins, neurofascin-186 (NF186), gliomedin, contactin and NCAM in 43% of GBS (*n* = 100) and 30% of CIDP (*n* = 50) patients (94). In addition, passive transfer of anti-gliomedin IgG to Lewis rat induced progressive neuropathy characterized by conduction defect and demyelination of spinal nerves (104). This has led the authors to suggest that myelin nodal proteins may play a role in induction of demyelination. Furthermore, clinical remission seen in these animals was parallel with the gradual decrease of IgG titers (94), suggesting that levels of antibody titers toward gliomedin could serve as a biomarker for disease remission.

Another study by Niezgoda et al. compared the level of serum NCAM in peripheral demyelinating neuropathy (PDN), axonal polyneuropathy (PN), non-inflammatory diabetic PN and healthy controls. In the study, Overall Neuropathy Limitation Scale (ONLS) and electrophysiological analysis comprises of motor and sensory studies were employed for clinical assessment. Significant increase in NCAM was seen among PDN group (*n* = 40 GBS, 29 CIDP, 11 Multifocal Motor Neuropathy) compared to other groups. In patients with PDN but not PN and non-inflammatory diabetic PN, serum NCAM levels had a high positive correlation to ONLS and negative correlation to motor conduction velocity. Thus, it has been concluded that NCAM detection could serve as a specific marker for peripheral nerve immune-mediated demyelination and sensitive marker for peripheral nerve involvement (95).

## Summary

Poor prognosis remained a dilemma in the management of the PDDs. Many people remain underdiagnosed or diagnosed when disease is already advanced. Moreover, the biomarkers that currently being used develop late in the disease process. Therefore, understanding the physiology of myelination and SC biology is vital to help further delineate the mechanisms involved in the pathogenesis of PDD. Autoantibodies against several types of gangliosides, phospholipids, glycoproteins, and nodal proteins have been shown to be present in numerous PDDs. However, their serum levels are yet to be correlated with a clinical course, and prognosis. A reliable biomarker should be sensitive and specific. Can peripheral autoantibodies that develop against the myelination-associated proteins or lipids in the early stage of PDDs be appropriate biomarker candidates? Future studies should explore this premise to discover time-sensitive biomarkers for early detection of PDDs.

## Author Contributions

KK and JK performed the literature search and drafted the manuscript. MY, RI, and SD reviewed and finalized the manuscript. The figures were designed by KK.

### Conflict of Interest Statement

The authors declare that the research was conducted in the absence of any commercial or financial relationships that could be construed as a potential conflict of interest.

## Footnotes

^1^Francine J Vriesendorp, MD Guillain-Barré syndrome in adults: Clinical features and diagnosis Post TW, ed. UpToDate. Waltham, MA: UpToDate Inc. http://www.uptodate.com (Accessed on May 09, 2018).

## Abbreviations

PDD: Peripheral Demyelinating Disease
SC: Schwann Cell
PNS: Peripheral Nervous System
CNS: Central Nervous System
P0: Protein Zero
PMP22: Peripheral Myelin Protein 22
MBP: Myelin basic protein
CSF: Cerebrospinal fluid
GBS: Guillain Barre Syndrome
GM-1: Monosialotetrahexosylganglioside-1
GD1a: Ganglioside GD1a
EBV, Epstein Barr virus. AIDP: Acute inflammatory demyelinating polyradiculopathy
AMAN: Acute motor axonal neuropathy
AMSAN: Acute motor sensory axonal neuropathy
LOS: Lipooligosaccharides
IVIG: Intravenous immunoglobulin
CIDP: Chronic inflammatory demyelinating polyradiculoneuropathy
NF155: Neurofascin-155
CNTN1: Contactin-1
MAG: Myelin Associated Glycoprotein
VEGF: Vascular endothelial growth factor
CMT: Charcot Marie Tooth
GDAP1: Ganglioside-induced differentiation-associated protein 1
MFN2: Mitofusin-2
GQ1b: Gangliosides Q1b
NLR: Neutrophil-lymphocyte ratio
PLR: Platelet-lymphocyte ratio
MRC: Medical Research Council
MLR: Monocyte-lymphocyte ratio
gAChR: Ganglionic nicotinic acetylcholine receptor
CNTN1/CASPR1: Contactin-1/contactin-associated protein-1
NCAM: Neuronal cell adhesion molecule
NavB1/2: Sodium channel at node of Ranvier ½
SGPG: Sulfoglucuronyl glycosphingolipid
ELISA: Enzyme-linked immunosorbent assay
P2: Protein 2
PC: phosphatidylcholine
PI: phosphatidylinositol
PG: phosphatidylglycerol
PS: phosphatidylserine
PE: phosphatidylethanolamine
PA: phosphatidic acid
MDL: Major denseline
PDN: Peripheral Demyelinating neuropathy
PN: Polyneuropathy
GJB1: Gap junction beta 1.
